# Industrial-Scale Application of Polymer Dewatering for Fine Tailings Disposal

**DOI:** 10.3390/ma18163872

**Published:** 2025-08-18

**Authors:** Rubén H. Olcay, Sayra Ordóñez, George E. Valadão, Francisco Patiño, Andréia B. Henriques, Iván A. Reyes, Julio C. Juárez, Mizraim U. Flores

**Affiliations:** 1 Departamento de Ingeniería Metalúrgica y Minas, Facultad de Ingeniería y Arquitectura, Universidad Arturo Prat, Avenida Arturo Prat 2120, Iquique 1110939, Chile; 2 Área Electromecánica Industrial, Universidad Tecnológica de Tulancingo, Tulancingo 43600, Mexico; sayraoh@hotmail.com; 3 Departamento de Ingeniería de Minas, Universidad Federal de Minas Gerais, Belo Horizonte 31270-901, Brazilabicalho@demin.ufmg.br (A.B.H.); 4 Área de Ingeniería, Ingeniería en Energía, Universidad Politécnica Metropolitana de Hidalgo, Tolcayuca 43860, Mexico; franciscopatinocardona@gmail.com; 5 Instituto de Metalurgia, Universidad Autónoma de San Luis Potosí, San Luis Potosí 78210, Mexico; alejandro.reyes@uaslp.mx; 6 Catedrático CONACYT, Consejo Nacional de Ciencia y Tecnología, Benito Juárez, Ciudad de México 03940, Mexico; 7 Área Académica de Ciencias de La Tierra y Materiales, Universidad Autónoma del Estado de Hidalgo, Carretera Pachuca-Tulancingo km 4.5, Carboneras, Mineral de la Reforma 42184, Mexico; jcjuarez@uaeh.edu.mx

**Keywords:** dewatering, laboratory scale, tailings, inert material

## Abstract

The treatment and safe disposal of mining tailings represent one of the main technical and environmental challenges in the contemporary mining industry. The present study aims to evaluate, at laboratory scale, three dewatering techniques applied to phosphate tailings: column thickener, hyperbaric filtration (horizontal filter press), and the direct application of a dewatering polymer. Based on the results obtained and the comparative analysis of Opex and Capex, the application of the dewatering polymer was selected for industrial-scale validation. The tailings sample presented an initial solids concentration of approximately 8.6% with very fine particle size, less than 70 microns. Under the best operating conditions for the aforementioned dewatering techniques, solids percentages by mass were obtained around ≈52% (thickening), ≈75% (filtration), and ≈40% (dewatering polymer). In all techniques, it was possible to obtain turbidity levels in the recovered water below 100 NTU, and a slight increase in the hardness of the overflows and filtrates was observed. According to the yield stress results, it was evident that the tailings were beginning to present characteristics of high-density slurry, paste, and cake with values of 40%, 48%, and 58% solids by mass, respectively.

## 1. Introduction

Today, water is a vital liquid for a comfortable life; it is indispensable for everyday life, cultivation, and industry; but now, there is an alarming scarcity of water worldwide, so many industrial processes require the rational use of this vital liquid [1,2,3]. Many companies are optimizing their processes to use the least amount of water possible; however, it is necessary to implement new processes in which the water that is discarded is recovered. Some of the industries that are using dewatering processes are paper, cement, polymer, ceramic, mining, and metallurgy [4,5,6,7,8,9,10]. The mining–metallurgical industry consumes millions of cubic meters of water every day, making it one of the industries that generates the most waste, and the waste water is rarely reused. Mining–metallurgical deposits have large quantities of water, so it is necessary to recover the greatest amount of this vital liquid [11,12,13,14,15,16,17,18,19,20]. Phosphate mineral purification is characterized by its high water consumption, as processing one ton of phosphate rock requires approximately 1.5 m^3^ of water [21,22,23,24]. The large quantities of water consumed in these processes generate fine phosphate tailings (FPTs), which can contain up to 90% of their weight in water [25,26]; therefore, the recovery of water from these tailings is essential to avoid the consumption of drinking water that would affect the surrounding populations. The management of mining and metallurgical waste is a major problem faced by all industries in this field, since thousands of tons of waste mineral are left in large tailings dams, which are very humid and have no economic value [27,28,29]. These non-commercial mining and metallurgical wastes must be stored; in addition, they are risky and represent a constant problem for the surrounding populations, since they are unstable due to the large amounts of water they contain [30]. Tailings are typically composed of water and complex gangue that is discarded after purification of the target mineral, including various clays that can be difficult to process [26,31,32]. There are many techniques for the treatment of fine-particle tailings, including bridging flocculation by the addition of water-soluble polymeric flocculants in gravimetric thickeners, which is a proven and effective process that achieves solid–liquid separation by increasing the settling rate of individual particles through the formation of larger aggregate structures [33,34,35,36]. There are many methods to dewater mining and metallurgical waste. Nowadays, many mineral processing and purification industries carry out this process using equipment that includes centrifuges, thickeners, dehydrating screens, vacuum filters, and pressure filters, which decreases the moisture content of the product and tailings [37]. It has been shown that the filtration process for iron ore tailings is possible using a filter press [38]. In addition to recycling a significant amount of water (approximately 10% of the total water consumption or approximately 80% of the filtration process), the main advantage of this method is the reduction in the volume of discarded tailings (approximately 63%).

Therefore, in this work, the dewatering efficiency of phosphate tailings was evaluated on a laboratory scale using different techniques such as column thickeners, hyperbaric filtration (horizontal filter press), and polymer dewatering agents. Polymer dewatering agents were selected for industrial-scale testing due to their efficiency and lower Capex and Opex compared with the other two techniques mentioned above. The tailings generated by the concentrator plant are approximately 250,000 tons/year; we aim to make the best possible use of water resources and available land in the dams and to achieve greater operational safety in the disposal of tailings with lower water content.

The main purpose of this study was to optimize tailings management at the industrial plant by evaluating various solid–liquid separation techniques at the laboratory scale. The goal was to select the most suitable technique for implementation in industrial-scale trials, aiming to achieve a tailings disposal with a higher solids concentration in the dam. This approach seeks to ensure the geotechnical stability of the tailings, maximize the use of the available storage space in the dam, increase the reuse of process water, and explore the potential for marketing the tailings as a byproduct, among other benefits.

## 2. Materials and Methods

### 2.1. Mineral Sample

The sample used in the various laboratory and pilot plant tests corresponds to the total tailings from the phosphate mineral processing plant located in the state of Minas Gerais, Brazil. The tailings to be dewatered come from the conventional and column flotation stages, which have an average solids content of around 8.6% (limiting variations under operating conditions range from 8% to 10% solids content).

The tailings were characterized physically and chemically using different analytical techniques. Density was determined using the simple pycnometer and the gas pycnometer methods. Particle size distribution was obtained using a Microtrac/MRB, Flow-Sync model (NY, USA). The chemical composition was analyzed by X-ray fluorescence (XRF) using a wavelength dispersive wavelength spectrometer (WDXRF), model ARL™ PERFORM’X—Thermo Scientific™ GEN-X 4200W, equipped with a rhodium tube (5GNf Rh 50 u) and an automatic X–Y manipulator (Labexchange, Burladingen, Germany). The specific surface area of the particles was determined using the Blaine apparatus (IBERTEST, Spain). All physical and chemical characterization tests were performed in the laboratories of the Department of Mining Engineering at the Federal University of Minas Gerais, Brazil.

The phosphate beneficiation process begins with the extraction of ore from an open-pit mine, followed by loading and hauling to the crushing and screening stage. Here, the ore is sorted by size to separate coarse fragments from fine ones (desliming). The fine fraction is sent directly to column flotation, while the coarse fraction undergoes grinding before entering conventional flotation. In both flotation methods, phosphate concentrate and tailings are produced. The tailings streams from both flotation stages are then combined and sent to the dam for final disposal.

**Figure 1:**
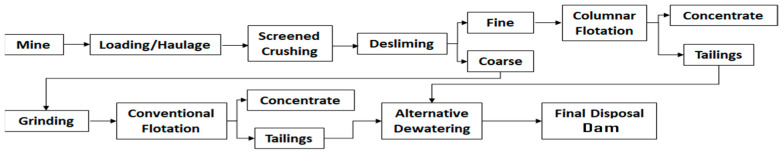
Overview of the phosphate ore processing plant.

Figure 1 represents an overview of the phosphate ore processing plant; in this image, each of the stages of the process that is carried out are shown.

### 2.2. Alternatives for Tailings Dewatering

**Figure 2:**
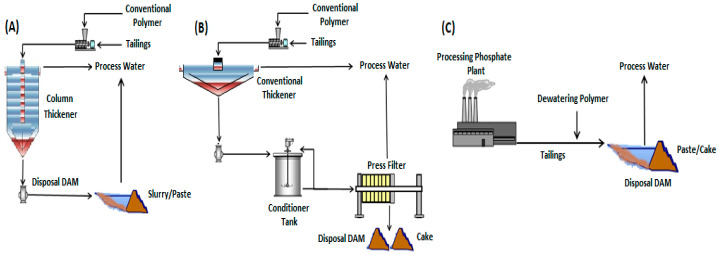
Overview of alternatives for tailings dewatering. (**A**) column thickener, (**B**) horizontal filter press, (**C**) direct application of dewatering polymer.

Among the different alternatives for tailings dewatering are thickening, using a column thickener, where a conventional polymer is added to the tailings beforehand to improve the operational performance of solid–liquid separation; filtration, through the use of a horizontal filter press, preceded by a pre-thickening of tailings solids before feeding the filter to increase process efficiency; and direct flocculation of the tailings, with the application of a dewatering polymer in the discharge line of the final tailings stream from the mineral processing plant. Figure 2 presents a diagram with the different dewatering alternatives for the final tailings disposal. 

### 2.3. Alternative Use of Column Thickener with Conventional Polymer

**Figure 3:**
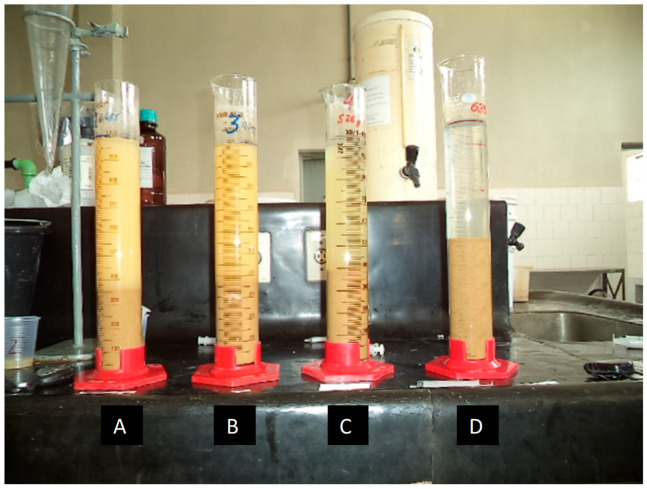
Conventional polymer screening tests: (**A**) 0 g/t, (**B**) 10 g/t, (**C**) 20 g/t, (**D**) 30 g/t (g/t: grams of polymer per ton of ore).

The tests carried out on the laboratory-scale column thickener replicated the most critical tailings mass concentration conditions typically employed in industrial operations, i.e., with a continuous feed of slurry at 8.6% solids by mass. The conventional polymer, previously selected by batch sedimentation tests in 1000 mL test tubes, was added. The concentration of the polymers evaluated was 0.1% *w*/*v*, and their selection was based on three main criteria: sedimentation rate, turbidity, and supernatant pH. Figure 3 shows the test tubes used in the conventional polymer selection process.

The column thickener used in this study consists of three acrylic modules: two with cylindrical geometry and one discharge module. Each module has a height of 0.6 m and an internal diameter of 0.1 m, reaching a total height of 1.8 m and a useful volume of 10 L. The system has a series of nozzles with an internal diameter of 0.01 m, spaced every 0.09 m, which facilitates continuous feeding, recirculation, and sampling of the material. The tailings are discharged (underflow) through the bottom of the equipment through a 45° cone, while the overflow is collected at the top through a 0.05 m internal diameter nozzle. The thickener system also includes a tank with an agitator, peristaltic pumps, and a floc generation reactor (FGR). The latter incorporates a helical mixer located before the slurry enters the thickener in order to promote particle aggregation and optimize solid–liquid separation.

**Figure 4:**
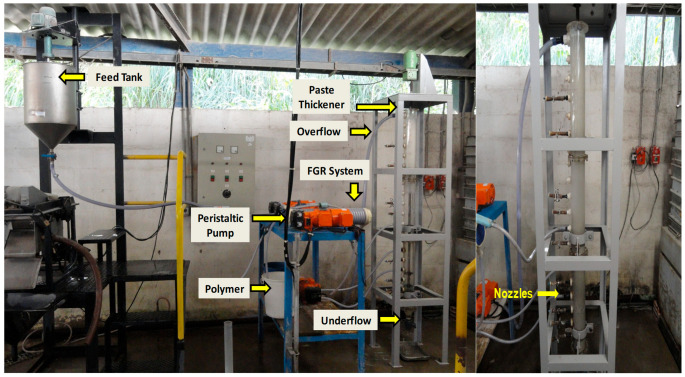
Column thickener for dewatering fine tailings [39].

It should be noted that this equipment corresponds to the one used in the study entitled, “Dewatering of Fine Tailings for Disposal in Dams Using a Column Thickener, Deep Cone Classifier, and Hyperbaric Filtration” [39]. The primary objective of the tests was to maximize solids concentration in the discharge and minimize overflow turbidity. Figure 4 presents an overview of the column thickener used in the dewatering process.

### 2.4. Alternative Use of Horizontal Filter Press

**Figure 5:**
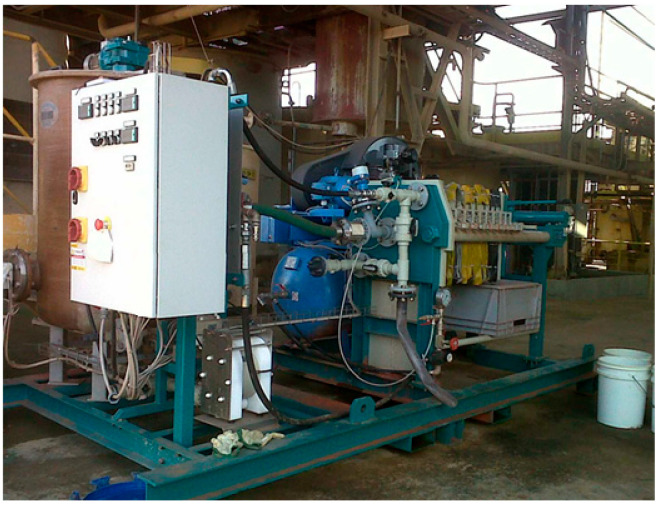
Horizontal filter press for dewatering fine tailings.

A horizontal filter press is a solid–liquid separation device widely used in industrial processes such as mining. Its main function is to remove liquid from a slurry (solid + liquid) using mechanical pressure. Its mode of operation consists mainly of the following steps: (i) Equipment preparation: The filter plates are closed by a hydraulic system to form airtight chambers. The condition of the filter cloths is checked so that there are no leaks in the valves or pipes. (ii) Slurry feeding: The pump drives the slurry from a tank into the filter chambers. The slurry enters through a central channel and is distributed to each chamber formed between two plates. (iii) Filtration: The liquid (filtrate) passes through the filter cloth, driven by the pressure of the pump. The solids are retained in the chamber, forming a cake. The filtrate is conveyed through internal channels to the outlets. (iv) Cake formation: The cake gradually forms as more solids are trapped. When the chamber is full, feeding is stopped. (v) Blowing or drying (optional): Compressed air or steam is injected to expel residual liquid and improve drying. A washing step may be included. (vi) Opening and unloading: Hydraulic pressure is released, and the plates are opened one by one. The cakes fall by gravity or vibration. They are collected for disposal or reprocessing. (vii) Cleaning and restarting the cycle: The cloths are cleaned manually or automatically if they are clogged. The equipment is inspected, and the process is restarted. Filtration tests were performed using a laboratory-scale recess-type filter press under different operating conditions to obtain different degrees of slurry dewatering. The parameters that were varied for performing the tests considered the slurry feed pressure, use of filtration aid, and pressing and drying times (cycle time). Figure 5 shows the equipment used in the laboratory tests.

### 2.5. Alternative Use of Dewatering Polymer

The use of dewatering polymers in the treatment of mining tailings has established itself as an efficient alternative for optimizing the management and disposal of these wastes. In laboratory tests aimed at selecting the dewatering polymer, 0.5 L of tailings with a solids content of 8.6% in mass were used. Following the manufacturer’s recommendations, the polymers evaluated (anionic and cationic) were prepared at a concentration of 0.25% (*m*/*v*).

**Figure 6:**
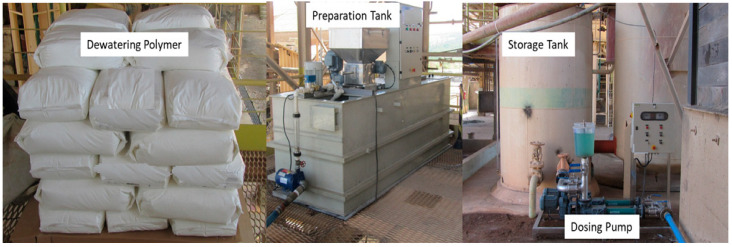
Dewatering polymer preparation and dosing system.

The mixing procedure consisted of manual stirring using two 0.5 L beakers, transferring the contents between them on three consecutive occasions to ensure homogeneous dispersion of the polymer in the slurry in a short period of time. The selection of the optimal polymer for the pilot tests was based on a comparative analysis of rheological and stability parameters such as yield strength, slump, and angle of repose obtained in each test. Figure 6 shows the equipment used for the preparation and dosing of the dewatering polymer during the pilot dewatering tests.

## 3. Results and Discussion

### 3.1. Characterization of Mineral Sample

**Figure 7:**
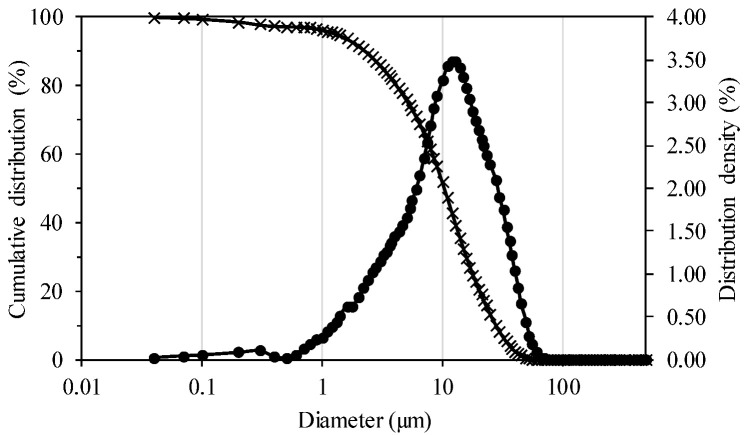
Granulometric distribution of mineral sample.

The specific weights of the samples were 2.84 g/cc (simple pycnometer) and 3.01 g/cc (gas), with a standard deviation of 0.12. For the purposes of calculations in the sedimentation, filtration, and dewatering tests, the value obtained by gas pycnometry was used. The size distribution of the mineral sample is presented in Figure 7, which shows a very fine granulometry, with 100% less than 70 µm and a mean size value of around 10.5 µm.

The chemical composition of the mineral sample was obtained by X-ray fluorescence, and the main results are presented in Table 1. A predominance of SiO_2_, Al_2_O_3_, CaO, and Fe_2_O_3_ compounds and, in smaller proportions, of P_2_O_5_ and MgO can be seen.

The chemical characterization of the mineral sample represents a waste with a low environmental impact, with a composition rich in SiO_2_, Al_2_O_3_, and CaO, suggesting significant potential for reuse in various industries such as construction, ceramics, and cement. Furthermore, the presence of components such as Fe_2_O_3_ and P_2_O_5_ indicates opportunities for the recovery of valuable elements, thus contributing to sustainability and the circular economy in the mining sector.

Regarding the specific surface area, it is presented in Table 2, using the BET and Blaine techniques. Both techniques presented a variation of less than 5% with an average equal to 0.916 m^2^/g.

The specific surface area of approximately 0.916 m^2^/g and the high concordance between BET and Blaine indicate that the tailings likely have a stable and consistent particle size distribution, which can be considered suitable for industrial applications. Furthermore, a high specific surface area can lead to increased reagent consumption, such as the use of filtration aids, polymers, collectors, etc.

### 3.2. Tests Using a Column Thickener Using Conventional Polymer

Table 3 presents the sedimentation rates obtained in 1000 mL test tubes with the different conventional polymers, cationic (CA), anionic (AN), and non-ionic (NI) types, with a dose of 50 g/t for their preliminary selection.

**Figure 8:**
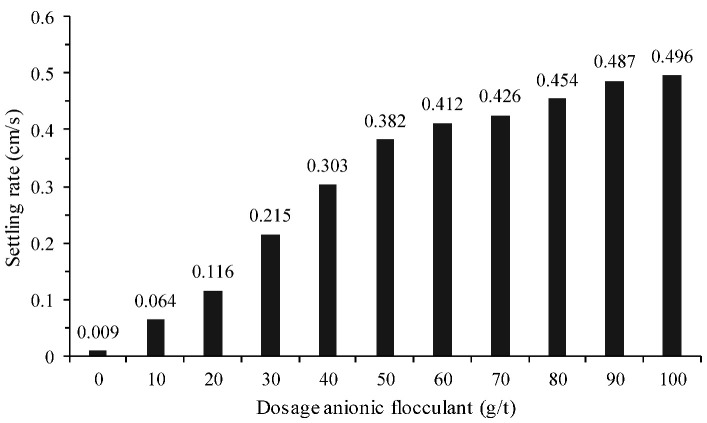
Tailings sedimentation rates as a function of the selected anionic polymer dosage (AF2 polymer).

Table 3 shows that, at a dosage of 50 g/t, anionic polymers (AF) achieved the highest sedimentation rates and relatively low turbidity (<100 NTU), clearly outperforming cationic and non-ionic polymers. This trend is consistent with previous studies, in which high-molecular-weight anionic flocculants performed better, promoting the formation of compact (dense) flocs and more efficient sedimentation for solid–liquid separation. The conventional polymer selected was AF2, which increased the sedimentation rate 42-fold compared with sedimentation without the use of polymer (0.009 cm/s). In turn, the turbidity of the obtained supernatant was less than 100 NTU, which, in principle, makes recirculation of the supernatant to the concentration plant feasible. Figure 8 shows the sedimentation rate for different doses of the selected polymer.

**Figure 9:**
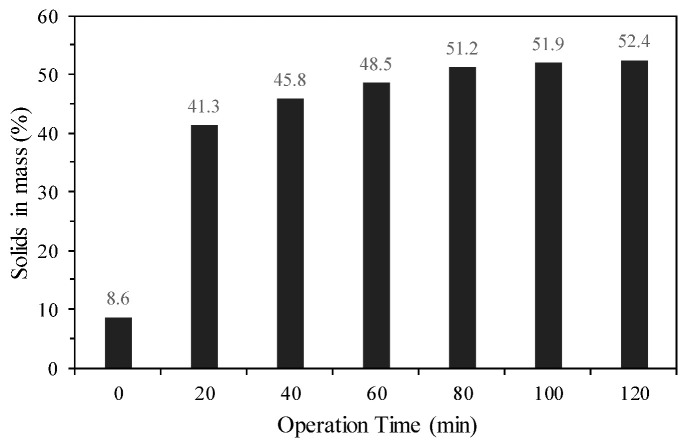
Percentages of solids by mass obtained at the discharge of the column thickener (AF2 polymer).

Figure 9 shows the mass solids concentrations obtained at the discharge of the column thickener over a total continuous operation time of 120 min with a dosage of 90 g/t of conventional anionic polymer (AF2) at a concentration of 0.1% *m*/*v* and a tailings feed of 8.6% mass solids (according to Figure 2A). The high dosage of conventional polymer selected may be due to the fine granulometry present in the tailings, since it has an average specific surface area of 0.916 m^2^/g.

As shown in Figure 9, the highest concentrations of solids by mass were observed after 80 min of continuous operation, with approximately ≈51% solids in mass, i.e., an approximately 6-fold increase in the percentage of solids by mass. After 120 min continuous operation, a maximum value of 52.4% was reached. According to measurements taken of the tailings’ yield stress at different solids concentrations, values of approximately 210 Pa were indicated, which classifies the tailings as a mineral paste [39,40,41,42]. It should be noted that the values obtained for supernatant turbidity under the indicated operating conditions were less than 100 NTU.

### 3.3. Tests Using a Horizontal Filter Press

**Figure 10:**
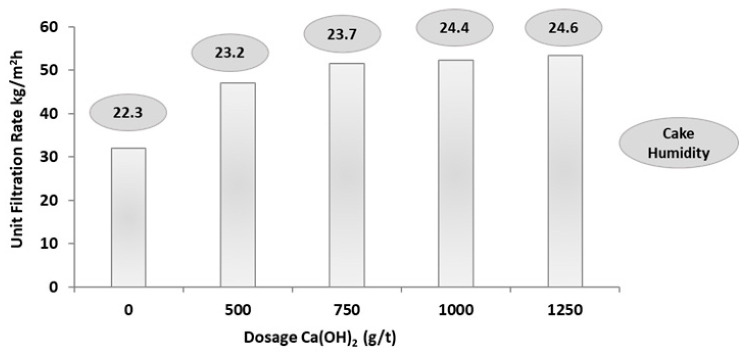
Unit filtration rate versus dosage of hydrated lime.

It should be noted than, for both the column thickener and the dewatering polymer tests, the feed slurries had a solids content of 8.6% solids in mass. Figure 10 shows the results obtained in the horizontal filter press under the most favorable operating conditions: the slurry fed to the filter plates had 20% solids in mass (conventional thickener discharge, according to Figure 2B), a feed temperature of 25 °C, and a chamber pressure of 7 bar, and compressed air was used for final drying of the cake. Hydrated lime was used as a filtration aid to reduce the presence of colloids in the filtrate. Under these conditions, cakes with a total moisture content of less than 25% were obtained. Although cakes with moisture contents less than 15% are considered optimal in industrial operations, this filtration efficiency was not achieved in the tests performed.

**Figure 11:**
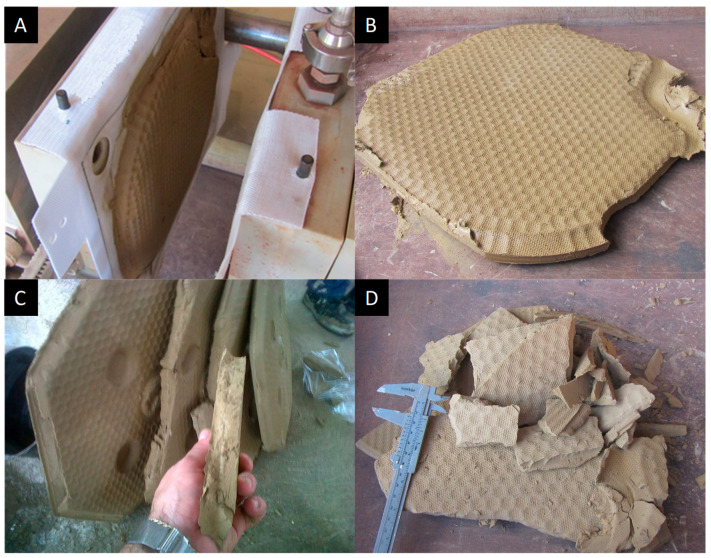
Cakes obtained in horizontal filter press tests ((**A**) filter plate, (**B**) filter cake, (**C**) cake thickness, (**D**) cake weighing).

In Figure 10, it is observed that to obtain a cake with values around 22% humidity without the use of hydrated lime, the filtration rate was approximately 30 kg/m^2^h. The best filtration rates were around ≈75% solids in mass with 50 kg/m^2^h, where the use of a filtration aid (hydrated lime) was required with doses from 500 g/t to 1250 g/t; the turbidity values of the supernatant in all the aforementioned tests with the use of filtration aid were less than 100 NTU. The filtration rates obtained were considered insufficient because the minimum expected for industrial operation is 200 kg/m^2^h. The lower filtration efficiency could be due to the high presence of fine particles in the tailings and the percentage of solids fed to the filter. The cakes obtained from the filtration tests are shown in Figure 11.

It should be noted that, in order to achieve higher production rates, a reduction in the efficiency of the dewatering process is expected, resulting in cakes with higher moisture contents. This is because conditions that favor higher filtration rates, such as the use of filtration aids and a lower degree of fine solids retention, tend to limit the final drying capacity of the cake.

### 3.4. Test Using Dewatering Polymer

**Figure 12:**
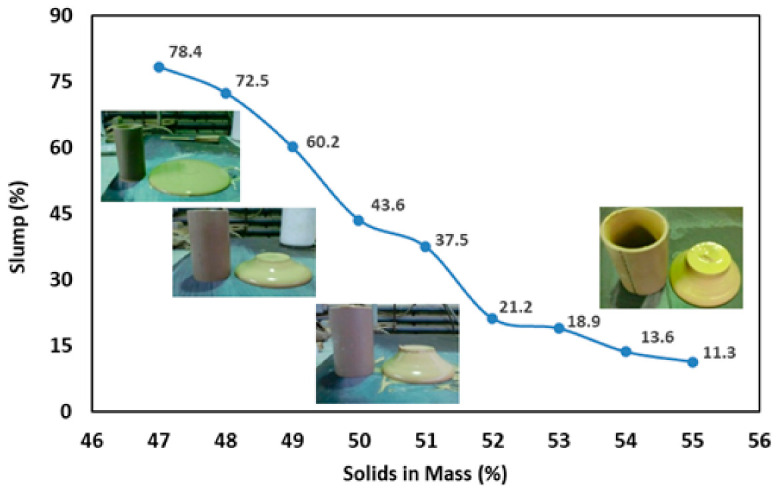
Tailings consistency for different percentages of solids by mass of the tailings.

**Figure 13:**
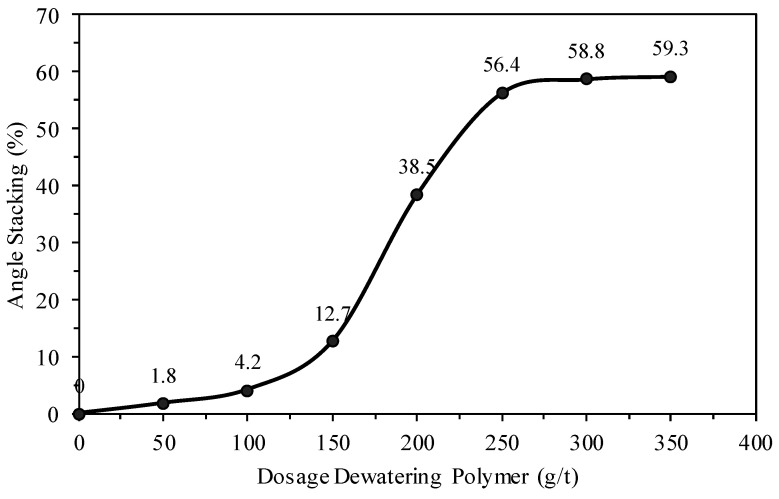
Tailings disposal angles for different dewatering polymer dosages.

**Figure 14:**
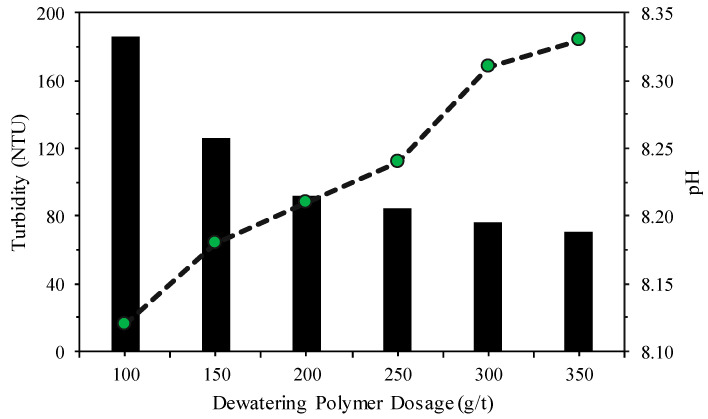
Turbidity and pH obtained in the supernatant of the condensed tailings.

The selection of the dewatering polymer was based primarily on parameters such as tailings consistency (slump), angle of repose, and supernatant turbidity, evaluated at different dosages of the dewatering polymer. Figure 12, Figure 13 and Figure 14 present the results obtained for the different tailings solids concentrations and the corresponding dosages of the selected polymer.

As observed in Figure 12, low tailings slump values were obtained, in the range of approximately ≈11% to ≈21%, for mass solids concentrations greater than 51%. These results demonstrate good tailings stability, typical of materials in the mineral paste state. According to Olcay et al. (2024), this state is characterized by a yield stress greater than ≈200 Pa. Additionally, recent research highlights that the combination of high solids concentration and the presence of fines promotes a significant increase in yield stress, reinforcing the classification of tailings as mineral paste [39].

According to Figure 13 and Figure 14, respectively, tailings disposal angles of over 35% were obtained with a 200 g/t (with a pH less than 8.35) dewatering polymer dosage and supernatant turbidity values of less than 100 NTU. These parameters are important in the study due to the improved utilization of the available pond volume for tailings disposal along with the recirculation of the supernatant to the concentration plant.

**Figure 15:**
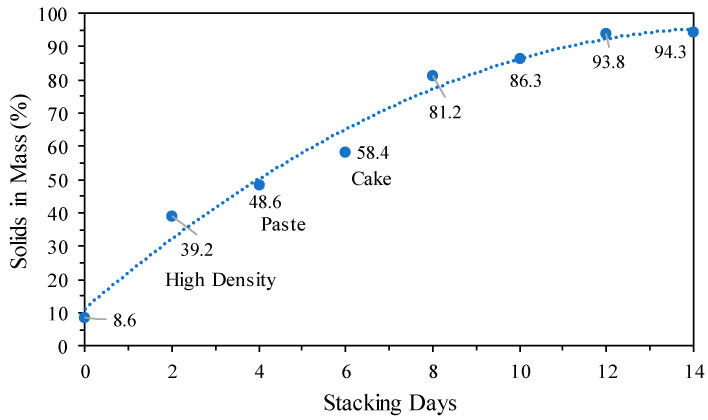
Dewatering of tailings with dewatering polymer as a function of time.

It should be noted that the solids mass percentages were calculated according to Equation (1). Figure 15 shows the evolution of tailings dewatering with the application of 200 g/t of dewatering polymer as a function of time (a total period of 14 continuous days) under ambient temperature conditions in the solid–liquid separation laboratory.

(1) $$\%Solids\;in\;mass=\frac{Mass\;of\;solids}{Mass\;of\;slurry}\times 100$$

It is observed in Figure 15 that initially, the tailings slurry with approximately 8.6% solids in mass (according to Figure 2C) released more than 75% of the water contained with the application of the dewatering polymer. It should be noted that the solids concentration in the slurry increased around 4-fold immediately and reached approximately 40% solids in mass on the second stacking day (for example: initially considering 86 g of solids and 1000 g of slurry, there is 8.6% solids in mass. For an 80% water loss from the slurry, the slurry mass is 268.8 g (86 g solids + 182.8 g water), which gives a solids percentage of around 32% solids in mass). From the second day of dewatering, the tailings presented rheological characteristics of slurry with high density; after the fourth and sixth days, paste and cake characteristics of the tailings were obtained, respectively.

To measure the yield stress, the vane method was employed using a Haake digital rheometer (Waters-instruments, model VT-550, New Castle, UK). The same procedure was applied to all samples, each containing different solid mass percentages (*w*/*w*). A four-blade vane-in-cup geometry was used, with each blade oriented at 90° relative to the adjacent one. The vane was immersed to a consistent depth in each sample, and the applied torque was increased progressively over approximately 300 s until a peak value was reached. Thereafter, the torque was gradually reduced until the measurement ceased.

**Figure 16:**
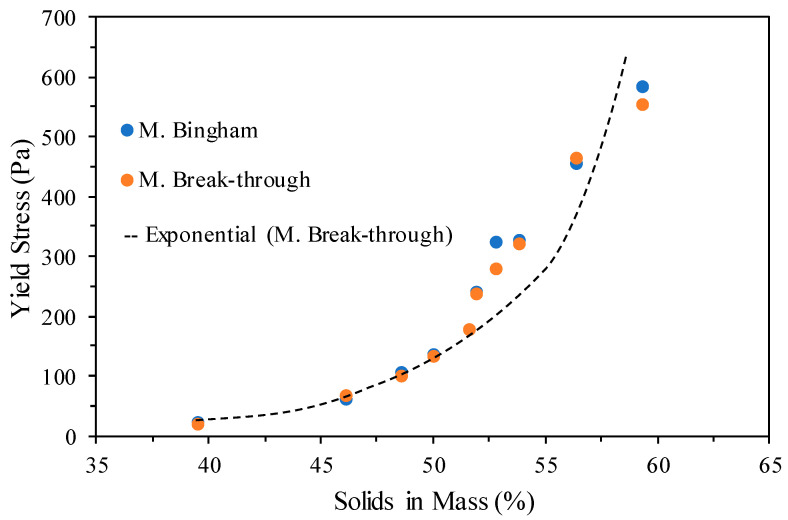
Yield stress versus percentage solids in mass of the tailings.

Figure 16 presents the yield stress measurements obtained by the Vane method with the Bingham and Breakthrough models for different percentages of solids by mass of the tailings.

As Figure 16 shows, the tailings have rheological characteristics of mineral paste and cake at around ≈48% (≈100 Pa) and ≈58% (≈500 Pa) of solids by mass, respectively, confirming the paste characteristics with values around 50% of solids by mass. Once the analysis of all the results obtained on a laboratory scale from the tailings dewatering by the different techniques was carried out, the dewatering polymer was selected to carry out the tests on an industrial scale, where aspects of investment costs and industrial operation (Capex and Opex) were also considered. Regarding Capex, the use of dewatering polymer involves a significantly simpler infrastructure (polymer dosing system, feed pumps, and simple piping), which results in a lower initial investment compared with the greater complexity and size of the equipment associated with a column thickening system (tall structures, mechanical systems, control instrumentation, etc.) and/or filter press (hydraulic structures, filter plates, washing systems, etc.). Additionally, the flocculant-based system can be implemented in smaller spaces (less area required), which reduces costs related to civil works, earthworks, foundations, and site preparation, among others. In the case of Opex, the use of polymer dewatering presents several advantages, such as lower energy consumption, since the process is based on flocculation and natural sedimentation. This significantly reduces the reliance on energy-intensive electromechanical equipment (e.g., high-pressure pumps and filter presses). In addition, the system requires fewer personnel due to its simple operation, a lower degree of automation and control, reduced need for specialized operators, and less-frequent maintenance. Maintenance costs are also lower, as the flocculant preparation system involves equipment with a low wear rate. In other words, more costly tasks such as filter plate maintenance, cloth cleaning, frequent replacement of filtering elements, or structural repairs to thickeners are eliminated.

### 3.5. Analysis of Supernatant and Filtrate Waters from Dewatering Tests

Table 4 presents the results of chemical analysis of the supernatant and filtrate waters generated under the best operating conditions for each tailings dewatering technique.

As observed in Table 4, the new process water is slightly alkaline, with small variations in pH that may be due to the application of the polymers and filtration aids used. Calcium and magnesium levels showed slight increases in the water obtained from the three dewatering techniques, possibly due to the presence of ultrafine particles (“colloids”), which generated an increase in aspects of water hardness. Turbidity values were considered acceptable and controlled for recirculation to the industrial process.

### 3.6. Industrial Test with Application of Dewatering Polymer

**Figure 17:**
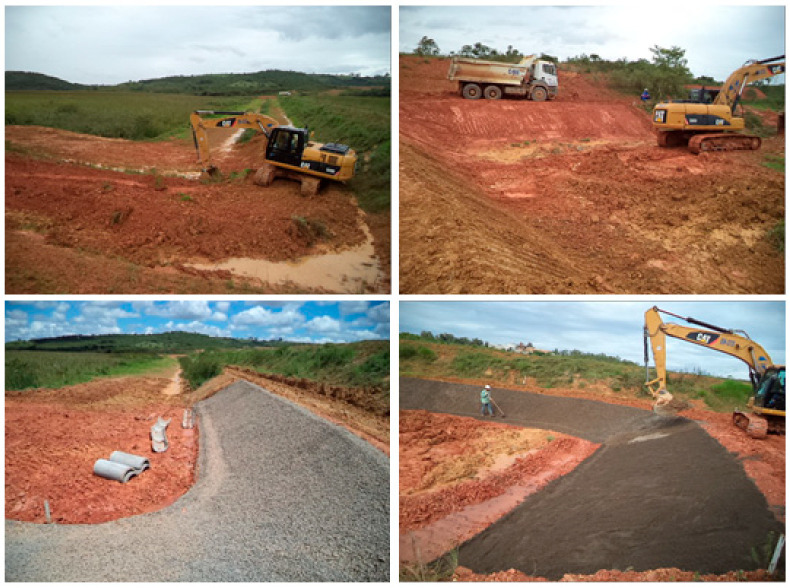
Preparing the ground for industrial testing.

The site preparation for the industrial test was carried out following a technical sequence that guaranteed controlled and safe conditions for the disposal of the treated tailings. This stage primarily included clearing and leveling of the designated area, ensuring a uniform surface free of obstacles. The containment slopes were then waterproofed by placing various layers of materials, including gravel, magnetite, and sand, culminating in the installation of a textile geomembrane. This configuration sought to prevent leaks, optimize surface drainage, and facilitate observation of the effects of the dewatering polymer under real-life operating conditions. Figure 17 shows the sequence of site preparation for the industrial test.

**Figure 18:**
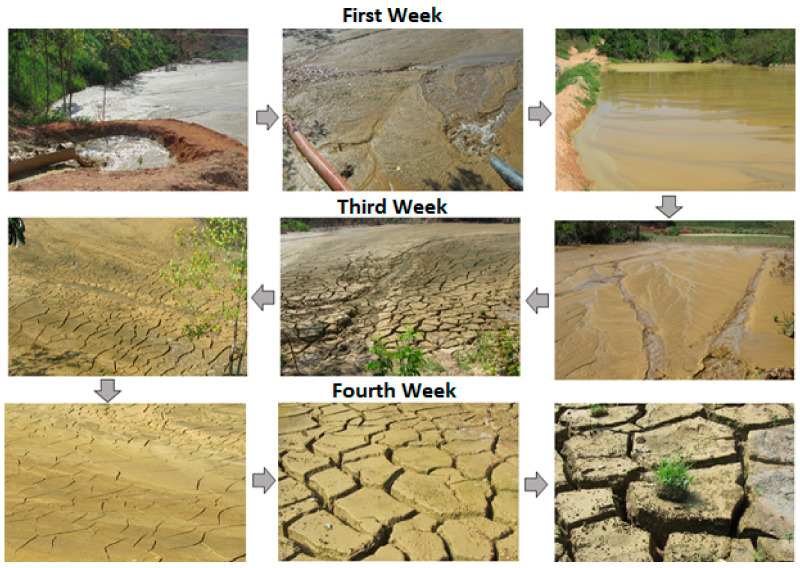
Visualization of tailings disposal with application of dewatering polymer.

During the industrial test, the dewatering polymer was applied to the tailings in an initial disposal stage that lasted for a week of continuous operation. As seen in Figure 18, the tailings’ response to the treatment was immediate and highly visible. From the very beginning, the formation of a disposal beach was evident, accompanied by runoff channels where water was significantly released.

After the tailings were suspended with the dewatering polymer, a natural dewatering phase began in the open air, lasting three to four weeks. During this period, the material continued to consolidate progressively, showing increasing formation of drying cracks, which allowed for improvements in the tailings’ structural consistency. These conditions facilitated the movement of operators and machinery over the already disposed material, enabling its removal using front-end loaders and trucks. This operational result not only freed up the area for further testing but also confirmed the effectiveness of the polymer under real-world field conditions, validating its use as a viable tool for improving tailings management.

## 4. Conclusions

The study conducted allowed for a comparative evaluation of three dewatering technologies applied to a fine-grained phosphate tailing through tests in a column thickener, hyperbaric filtration, and the application of the dewatering polymer. The phosphate tailings sample presented a very fine particle size distribution, with a particle size of less than 70 microns, which somewhat facilitated the formation of high-density slurry and mineral paste with relatively low solids by mass percentages. Tests in the column thickener yielded discharge concentrations of approximately ≈52% solids by mass for periods exceeding 80 min of continuous operation, exhibiting rheological characteristics of a mineral paste (≈200 Pa). In the case of hyperbaric filtration with the horizontal filter press, cakes with moisture levels around ≈25% were obtained, but not with the high production rates required at an industrial level. The use of a dewatering polymer enabled a high release of the water contained in the slurry, with values above ≈75%. It should be noted that the results mentioned with each technique were obtained under the best operating conditions found. The technical and economic analysis (Capex and Opex) demonstrated that the industrial application of the dewatering polymer represents a viable, efficient, and rapidly implemented alternative for tailings management under real-world operating conditions. The industrial trial validated its effectiveness, presenting satisfactory results and demonstrating an immediate response from the treated tailings, the formation of stable beaches, and the progressive consolidation of the disposed material. This included the production of mineral paste and cake starting in the third and fourth weeks of tailings disposal in the dam, facilitating their handling and subsequent removal. From a sustainability perspective, disposing of tailings in a state of higher solids concentration allows for increased process water recovery, optimized use of available space in dams, and improvement in the geotechnical safety of containment structures. Furthermore, the physical and chemical characterization of the tailings demonstrated its potential to be considered as an input in other industries, such as construction, ceramics, and cement manufacturing, opening up new opportunities within the mining circular economy. It is recommended that pilot and industrial-scale studies continue to be conducted to comprehensively valorize tailings as well as to evaluate the long-term stability of the material disposed of with the dewatering polymer under different climatic and geotechnical scenarios.

## Figures and Tables

**Table 1:** Chemical composition of the mineral sample by X-ray fluorescence.

Component	%	Component	%
P_2_O_5_	2.34	SiO_2_	60.59
Fe_2_O_3_	5.27	Al_2_O_3_	14.83
MgO	1.18	CaO	9.12

**Table 2:** Calculation of specific surface area.

Technique Used	Specific Surface Area (m^2^/g)
BET	0.937
Blaine	0.894

**Table 3:** Sedimentation velocities (cm/s) and turbidity (NTU) of the supernatant for cationic (CF), anionic (AF), and non-ionic (NIF) polymers.

Settling Rate/Turbidity Cationic Polymer	Settling Rate/Turbidity Anionic Polymer	Settling Rate/Turbidity Non-Ionic Polymer
CF1: 0.142/187	AF1: 0.287/74	NIF1: 0.112/154
CF2: 0.217/241	AF2: 0.382/86	NIF2: 0.181/179
CF3: 0.259/284	AF3: 0.311/83	NIF3: 0.105/143

**Table 4:** Analysis of supernatant and filtrate waters obtained under the best operating conditions for each tailings dewatering technique.

Type of Water	pH	Ca (ppm)	Mg (ppm)	Turbidity (NTU)	Hardness
Water before the process overflow	8.1	7.2	3.6	<100	31.2
Column thickener	8.5	11.9	4.9	<100	56.5
Filtration (hyperbaric filter)	9.2	12.1	5.8	<100	60.9
Polymer dewatering overflow	8.3	10.4	4.7	<100	51.3

## Data Availability

The original contributions presented in this study are included in the article. Further inquiries can be directed to the corresponding authors.
